# A Tough, Water-Resistant, High Bond Strength Adhesive Derived from Soybean Meal and Flexible Hyper-Branched Aminated Starch

**DOI:** 10.3390/polym11081352

**Published:** 2019-08-14

**Authors:** Yi Zhang, Jieyu Zhang, Mingsong Chen, Jing Luo, Sheldon Q. Shi, Qiang Gao, Jianzhang Li

**Affiliations:** 1 Beijing Key Laboratory of Wood Science and Engineering & MOE Key Laboratory of Wooden Material Science and Application, Beijing Forestry University, Beijing 100083, China; 2 College of Material Science and Engineering, Nanjing Forestry University, 159 Longpan Road, Nanjing 210037, China; 3 College of Engineering Department of Mechanical and Energy Engineering, University of North Texas, 3940 North Elm Street, Suite F101P, Denton, TX 76207-7102, USA

**Keywords:** soybean meal‐based adhesive, hyper-branched starch, crosslinking density, water resistance, toughness

## Abstract

Soybean meal (SM)-based adhesive exhibited a great potential to replace petroleum-derived ones to alleviate the energy crisis and eliminate carcinogenic formaldehyde. However, the bad water resistance (caused by low crosslinking density) and inherent brittleness of SM adhesive severely hindered its application. However, improving crosslinking density is generally accompanied by a toughness reduction of the adhesive. Herein, we developed a flexible long-chain starch with a hyper-branched structure (HD), and incorporated it with SM and a crosslinking agent to prepare a novel SM adhesive. Results showed that this adhesive exhibited both excellent water resistance and enhanced toughness. The wet bond strength of plywood fabricated using this adhesive was 354.5% higher than that of SM adhesive. These achievements are because introducing HD with hyper-branched groups enhanced crosslinking density, while HD’s flexible long-chain structure improved toughness of the adhesive. This HD can promote the development of tough and hydrophobic bio-based composites.

## 1. Introduction

Formaldehyde-based adhesives, play a dominant role in wood-based panel manufacturing, as they exhibit a high bond strength and low cost [1]. However, application to wood-based panels creates indoor pollution concerns due to continuous formaldehyde release [2]. Moreover, the raw materials required to synthesize these adhesives rely on unsustainable, petroleum-based resources [3]. As such, the development of environmentally friendly adhesives that are nontoxic and renewable is important from both a pollution control and energy resource perspective [4].

Soybean meal (SM), a byproduct after oil extraction from soy, has been used in the manufacture of bio-based wood adhesives for decades. The active groups in SM, such as –NH_2_, –COOH, and –OH, can form hydrogen bonds with –OH in the wood to produce a bonding effect [5]. However, these hydrogen bonds are weakened with moisture, resulting in a SM adhesive with poor water resistance and low bond strength, thus the plywood fabricated using these adhesives cannot meet the strength requirement for indoor use [6]. In order to improve the performances of SM adhesive, the most effective method is crosslinking agent modification; particularly epoxides, such as ethylene glycol diglycidyl ether [7], glycidyl methacrylate [8], and 5,5-dimethyl hydantoin epoxide [9]. During curing, the epoxy group reacts with the active groups in the SM, improving the adhesive’s crosslinking density and thus increasing water resistance and bond performance of the resultant plywood. However, increasing crosslinking density inevitably causes the increase of adhesive brittleness, which will decrease the adhesive’s impact resistance and result in a low bonding performance [10]. Therefore, improving crosslinking density and toughness of the adhesive simultaneously is a huge challenge.

In our previous research, we found that creating a hyper-branched crosslinking structure significantly increases the crosslinking density of the adhesive [11]. Based on the above fact, in this study, we design a flexible long-chain biopolymer with hyper-branched structure and exploit it to develop a novel high-performance SM adhesive that features highly crosslinking structure and excellent toughness. Specifically, long-chain dialdehyde starch (DAS) was acidified and heated to release the reactive aldehyde groups. The optimized DAS was grafted with hyper-branched polyamide (HBPA) to prepare a hyper-branched amino starch (HD) via a Schiff base reaction. The effects of grafting modification on the starch were assessed via determining the chemical structure, grafting rate, and thermal degradation behaviors. Subsequently, the HD was blended with SM in the presence of crosslinking agent-triglycidyl isocyanurate (TGIC) to generate a novel SM adhesive. The functions, toughness, morphological property, residual rate, and moisture uptake of the prepared adhesives were investigated to explain the strengthening mechanisms. Three-ply plywood bonded with each adhesive was prepared to measure the shear strength according to Chinese national standards (GB/T 9846.3-2004). To the best of our knowledge, this is the first time to explore whether a long-chain biomaterial with hyper-branched structure improves both crosslinking density and toughness of the thermosetting adhesive and develops a bio-based adhesive with a high bond performance and excellent water resistance. Moreover, the resultant HD, with its high-content functional groups, can also be employed to prepare self-healing hydrogels or enhance other biomaterials, which will enormously expand the scope of its applications.

## 2. Materials and Methods

### 2.1. Materials

Soybean meal, namely SM, (47% soy protein), is supplied by Xiangchi Grain and Oil Co., Ltd. (Binzhou, China). Dialdehyde starch (DAS) (90% aldehyde content) was purchased from Jinshan denatured starch Co. Ltd. (Taian, China). The molecular weight of DAS was measured as shown in Figure S1 (Supplemental Files). Specially, the DAS’s weight-average molecular weight was 4934 D. The crosslinking agent, i.e., triglycidyl isocyanurate (TGIC), is a triazine epoxy compound derived from epichlorohydrin and isocyanuric acid [12]. The TGIC, diethylenetriamine, succinic anhydride, and hydrochloric acid were all provided by Beijing Lanyi Chemical Products Co., Ltd. (Beijing, China). The poplar veneers were obtained from Haobo timber processing factory (Linyi, China).

### 2.2. Preparation of HBPA

Succinic anhydride (8.0 g) and diethylenetriamine (10.3 g) were mixed and then stirred at 140 °C for 3 h to obtain a homogenous mixture. The crude HBPA was dissolved in dimethylacetamide to remove the unreacted residues. Then the obtained suspension was centrifuged for 5 min at 8000 rpm. The collected precipitate was oven-dried at 105 °C to obtain a constant weight.

### 2.3. Improvement of the Aldehyde Group Content in DAS

The aldehyde groups in DAS generally exist in the form of hemiacetals or acetals, rather than in free form [13], and can be destroyed under heating and acidic or alkaline conditions [14]. Specifically, DAS (2 g) was mixed with 70 g of distilled water, then heated at 70 °C for 10 min. The solution pH was adjusted to 5 using 3.65% hydrochloric acid solution. The mixture was continuously stirred for 20 min to form gelatinized DAS.

### 2.4. Graft Copolymerization of HBPA onto DAS

HBPA (3.0 g) was introduced into the gelatinized DAS. Next, the suspension was maintained at 70 °C and stirred for 20 min to form a crude graft copolymer (HD). The crude HD was dissolved in methanol and then re-precipitated with acetone. Finally, the suspension was washed and filtered to collect the precipitate. The resulting precipitate was freeze-dried to obtain pure HD.

### 2.5. Characterization of DAS, HBPA, and HD

The sample’s molecular weight distribution was tested via gel permeation chromatography (GPC), which was conducted using a Waters GPC 1515 instrument (WATERS Company, Milford, MA, USA). The mobile phase was dimethyl sulfoxide. The injection volume was 10.00 μL and the flow rate was 1 mL/min. 

In order to quantitatively assess the degree of grafting, the original and grafted DAS samples were analyzed using ^1^H NMR. Terephthalic acid disodium salt was used as an internal standard substance. The samples were prepared as follows: Sample 1: 18.73 mg of HD and 2.81 mg internal standard were completely dissolved into D_2_O; Sample 2: 3.96 mg of DAS was dissolved in dimethyl sulfoxide (CD_3_)_2_SO; and Sample 3: 28.39 mg of HBPA and 2.15 mg terephthalic acid disodium salt was dissolved in D_2_O. The 300 MHz ^1^H NMR spectra of these samples were obtained using an ECS-400 spectrometer (JEOL RESONANCE Inc., Tokyo, Japan) with a 3.0 s acquisition time.

The grafting rate (G) was calculated using the following equations [15]:

(1) $$n_{1}=(A_{1}/A_{0-1})\times (m_{0-1}/M_{0})$$

(2) $$n_{2}=(A_{2}/A_{0-2})\times (m_{0-2}/M_{0})$$

(3) $$G=(n_{2}/n_{1})\times m_{1}/m_{2}$$

where *n*_1_ and *n*_2_ are the concentration of amino groups in the HBPA and HD, respectively; *M*_0_ represents the molar mass (g mol^−1^) of the terephthalic acid disodium salt; *A*_1_ and *A*_2_ represent NMR peak areas of the amino groups in HBPA and HD, respectively; *A*_0-1_ and *A*_0-2_ are the peak areas of the internal standard in HBPA and HD, respectively. The weights of HBPA and HD are labeled *m*_1_ and *m*_2_ (g), respectively.

The samples’ thermal stability was evaluated by a TA Q50 (WATERS Company, Milford, MA, USA). Samples with a weight of 6 ± 0.1 mg were scanned while being heated. The temperature ranged from 25 to 600 °C. The heating rate was 10 °C min^−1^. All experiments were performed in an N_2_ environment, and the samples’ weight change was recorded every 10 degree temperature increase.

### 2.6. Preparation of the HD-Modified SM Adhesive

Adhesive 1 was synthesized by adding 30 g of SM to 70 g of distilled water, then stirring at room temperature for about 30 min to obtain SM adhesive. Adhesive 2 (SM/TGIC) was formulated by introducing 2 g of TGIC into the SM adhesive mixture and stirring for 30 min. Adhesive 3 (SM/TGIC/DAS-5) was developed by adding 5 g DAS into adhesive 2, then mixing for about 30 min. The SM/TGIC/HD adhesives were prepared by dissolving 2.5, 5, or 7.5 g of HD into the SM/TGIC adhesive, followed by 30 min of stirring. The resultant adhesives were referred to as adhesive 4, 5, and 6, respectively. The corresponding adhesive formulations are listed at Table S1 (Supplemental Files).

### 2.7. Characterization of the Adhesive Samples

#### 2.7.1. Attenuated Total Reflectance-Fourier Transform Infrared Spectroscopy (ATR-FTIR)

The functional groups and structures of DAS, HBPA, HD, and the resultant SM adhesives were measured using a Nicolet 6700 ATR-FTIR spectrometer (Nicolet Instrument Corporation, Madison, WI, USA). The scanning region ranges were 4000~400 cm^−1^ with 32 scans. The resolution was 4 cm^−1^.

#### 2.7.2. Toughness Evaluation 

The toughness was visually assessed by observing the surface morphology of the adhesives [16]. Every adhesive solution covered a glass slide surface evenly. Next, the coated glass slide was transferred to a 120 ± 2 °C oven, to simulate the curing process of adhesives. After curing, the adhesives were cooled at room temperature. Finally, the pictures of these cured adhesive layers were recorded by a digital camera (Sony/DSC-HX400, Tokyo, Japan).

#### 2.7.3. Hydrolytic Stability

The adhesive was cured at 120 ± 2 °C, until it reached a stable mass (*m*_0_). This adhesive was then immersed in water at room temperature for 24 h. Next it was transferred to an oven (105 ± 2 °C) to obtain a stable mass (*m*_1_). The adhesive’s residual rate was calculated according to Equation (4), and each result is the average of six parallel measurements.

(4) $$\mathrm{Residual}\;\mathrm{rate}\;\mathrm{value}\;(\%)\;=\;m_{1}/m_{0}\;\times \;100\%$$

#### 2.7.4. Water Uptake Test

The completely cured adhesive was put into a constant-temperature constantly wet box. The parameters were set to 50 °C and 80% humidity. The adhesives’ mass was recorded every 2 h until it achieved a stable mass (*w*_1_). The water uptake value was obtained according to Equation (5), and every result is the average value of six parallel tests.

(5) $$\mathrm{Moisture}\;\mathrm{uptake}\;\mathrm{value}\;(\%)\;=\;(w_{1}-w_{0})/w_{0}\;\times \;100\%$$

where *w*_0_ is the adhesive’s initial weight.

#### 2.7.5. Scanning Electron Microscopy (SEM)

The adhesive’s fractured cross-section was examined by an SEM instrument (Hitachi S-3400N, Ibaraki, Japan). Before the test, the fractured surface of the adhesive was coated using an Au/Pd film (10 nm).

#### 2.7.6. Preparation and Measurement of Plywood

Three-ply plywood was fabricated using the proposed SM-based adhesives for the performance evaluation of adhesives. Firstly, 180 g/m^2^ adhesive was coated on the veneer (just one side). The hot-press process was conducted at 120 °C, under 1.0 MPa, for 6 min subsequently. After standing at room temperature for 12 h, every plywood was sawn into six battens (25 mm × 100 mm). Next, these battens were soaked in water (63 ± 2 °C) for 3 h, according to Chinese National Standards (GB/T 17657–2013). Subsequently, these soaked battens were put in air and cooled naturally for 10 min. After that, they were stretched with a 20.0 mm min^−1^ operating speed for shear strength testing. Specifically, the strength was obtained using Equation (6), note that six replicates per adhesive were measured, and the average was reported.

(6) $$\mathrm{Wet}\;\mathrm{shear}\;\mathrm{strength}\;(\mathrm{MPa})\;=\frac{\mathrm{Tension}\;\mathrm{Force}\;(N)}{\mathrm{Bonding}\;\mathrm{Area}\;(\mathrm{mm})}\times 100\%$$

## 3. Results and Discussion

### 3.1. Analysis of DAS/HD

**Figure 1:**
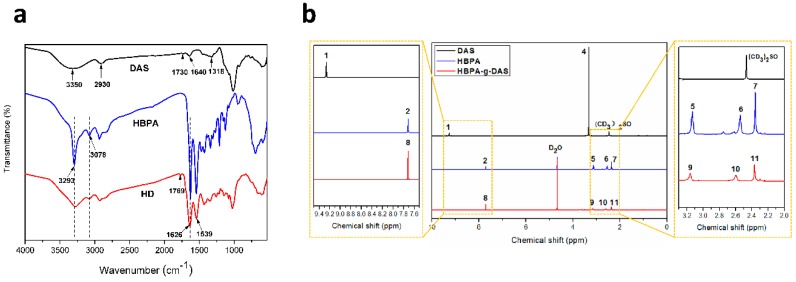
(**a**) FTIR and (**b**) ^1^H NMR spectra of dialdehyde starch (DAS), hyper-branched polyamide (HBPA), and hyper-branched aminated starch (HD).

**Scheme 1:**
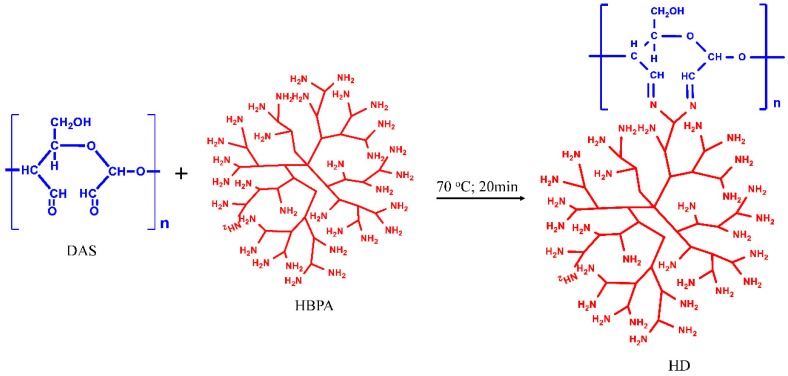
The grafting mechanism of HD.

The DAS, HBPA, and HD FTIR spectra are shown in Figure 1a. With respect to the DAS spectrum, the characteristic peak of aldehyde group appeared at 1730 cm^−1^ [17]. This peak exhibited a weak intensity because the aldehyde groups formed hemiacetals and acetals. Characteristic dialdehyde stretching peaks appeared at 1318 and 2930 cm^−1^. This result is consistent with Kamoun’s finding [18]. The peak around 3350 cm^−1^ corresponded to the vibration of hydroxyl. In addition, the peak at 1640 cm^−1^ was identified as tightly bound water in DAS [19]. In the HBPA spectrum, the two sharp peaks (3293 and 3078 cm^−1^) were ascribed to –NH_2_ stretching vibration [20]. The synthesis process of HBPA is shown in Scheme S1 (Supplemental Files). Regarding the HD spectrum, the peak belonging to the C=O groups (1730 cm^−1^) gained intensity and blue-shifted from 1730 to 1769 cm^−1^. This shift resulted from hemiacetals and/or acetals in the DAS being hydrolyzed after the acid and heat treatment and, subsequently, transforming additional reactive aldehyde groups. When DAS was grafted to form HD, two new sharp peaks were observed, corresponding to the vibration and hydrogen bond association of –NH_2_ [21]. Compared with DAS, a new peak (1539 cm^−1^) appeared in the HD spectrum, which corresponded to the C=N groups [22]. These data verified that HD was synthesized successfully through a Schiff base reaction, and this finding is consistent with results reported by Liu [23]. The aldehyde release and DAS grafting mechanism are shown in Scheme S2 (Supplemental Files) and Scheme 1.

The ^1^H NMR spectra of DAS, HBPA, and HD were evaluated to determine the structural changes to DAS during the grafting process. As shown in Figure 1b, DAS was analyzed by dissolving it in (CD_3_)_2_SO. The solvent’s proton signal was observed at 2.47 ppm [24]. The characteristic peak located at 9.30 ppm (peak 1) in the DAS spectrum was ascribed to the aldehyde groups’ proton signal, whereas the high-intensity signals at 3.05–3.75 ppm were attributed to the DAS H proton signals [25]. For the HBPA’s spectrum, the proton signals of the internal standard and solvent (D_2_O) were observed at 7.71 ppm (peak 2) and 4.67 ppm, respectively [26]. A strong proton signal appeared at 2.36 ppm (peak 7), which reflects a high concentration of N–H in the HBPA [27]. The signal at 3.13 ppm (peak 5) belongs to the H protons in the –CH_2_ of NH–CH_2_, NH_2_–CH_2_, and N–CH_2_ bonds, and the peak at 2.54 ppm (peak 6) is related to the H protons in the –CH_2_ of C=O–CH_2_ molecule. The structure of HBPA is shown in Scheme S1. After HBPA was grafted on the DAS, the typical peak of the aldehyde groups disappeared in the HD spectrum. In contrast to the DAS ^1^H NMR spectrum, two new signal peaks were observed at 2.60 ppm (peak 10) and 2.37 ppm (peak 11) in the HD, which were attributed to–CH_2_ and –NH_2_ of the HBPA. These findings confirmed that HBPA was grafted successfully onto the DAS backbone by reacting with the aldehyde groups in the DAS. The grafting was quantified by calculating the ratio of N–H to HD, and the grafting rate was computed as 75.8%; which is approximately 18% higher than the maximum grafting rate reported in Kouassi’s study [28]. The data used to calculate the grafting rate are listed in Table 1.

**Figure 2:**
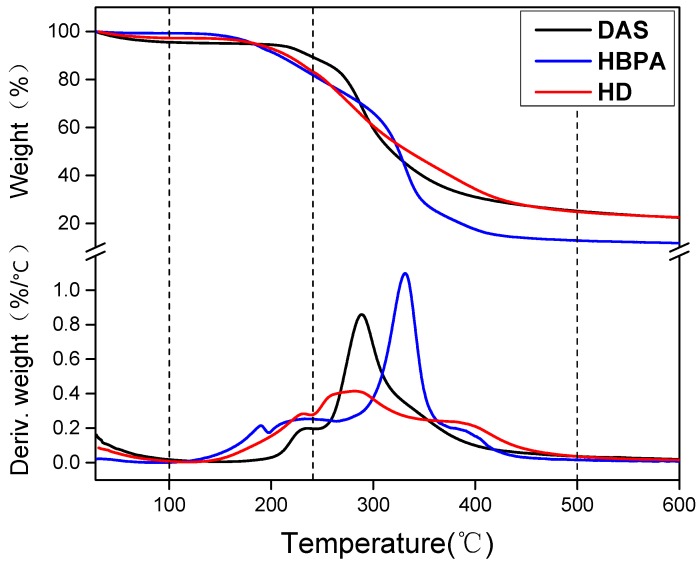
Thermogravimetric (TG) and derivative thermogravimetric (DTG) curves of DAS, HBPA, and HD.

Figure 2 shows the TG and DTG curves of DAS, HBPA, and HD. For DAS, mass loss in thermal degradation covers three stages. In stage I, from 28 to 100 °C, the evaporation of adsorbed water caused a weight loss of 12.2%. Stage II (100~250 °C) corresponded to the destruction of the DAS’s crystal structure and exhibited a degradation peak at approximately 232 °C on the DTG curves [23]. Stage III (250 ~500 °C) was characterized by decomposition of the DAS backbones, a peak at approximately 288 °C, and weight loss of 62.4%. In the HBPA DTG curves, the second stage is attributed to small-molecule decomposition in the hyper-branched cavity; and the branched side chains of HBPA were cleaved in the stage with a peak at 190 °C. In the third stage, HBPA exhibited a weight loss of 88% with a degradation peak (331 °C), which was caused by degradation of the main chain. After HBPA grafting, the weight loss of HBPA–*g*–DAS was 112% higher than that of DAS in the second stage, which was due to instability with the grafted amino groups. In the third stage, the HD DTG curve features only one peak at 283 °C. These results further confirmed that the grafting reaction between DAS and HBPA occurred and formed a new structure, agreeing with FTIR result. Moreover, HD degradation rate clearly decreased when compared with that of DAS, indicating that grafting HBPA onto the DAS improved the DAS’s thermo stability.

### 3.2. Analysis of Adhesive Samples

**Figure 3:**
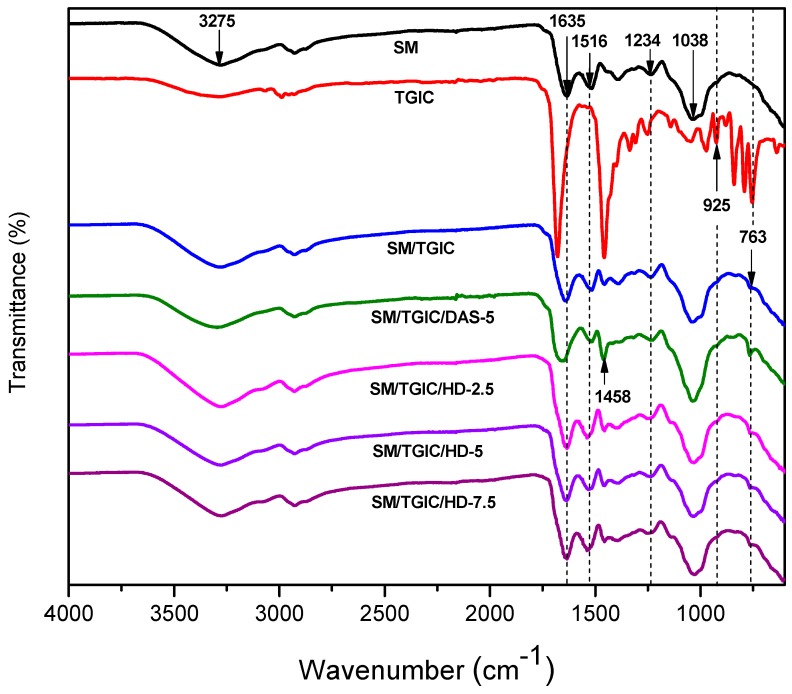
The attenuated total reflectance-fourier transform infrared spectroscopy (ATR-FTIR) spectra of the resulting adhesives.

**Scheme 2:**
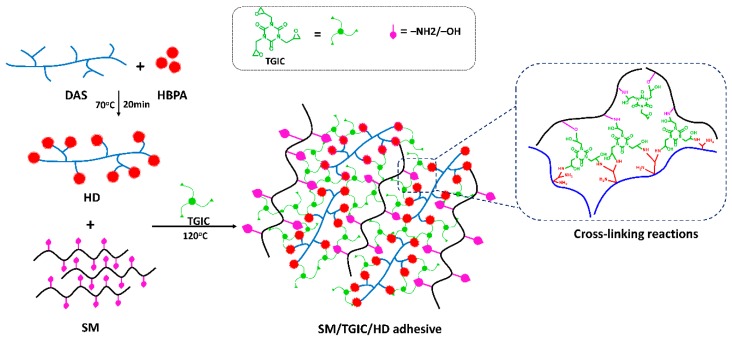
Crosslinking reaction in soybean meal (SM)/TGIC/HD adhesives.

The resulting adhesives’ ATR-FTIR spectra are displayed in Figure 3. For the SM spectrum, three characteristic absorption peaks at 1635, 1516, and 1234 cm^−1^ corresponded to amide I, II, III bands, respectively, i.e., C=O, N–H, and C–N/N–H bonds. Whereas the broad peak around 3275 cm^−1^ belongs to the N–H and O–H stretching. In the TGIC spectrum, the typical peak (925 cm^−1^) correspond to the epoxy group. The peak (764 cm^−1^) belongs to TGIC’s nitrogen-containing heterocyclic ring. When adding TGIC into the SM, the TGIC’s characteristic absorption peak (764 cm^−1^) appeared in the cured adhesives, suggesting the TGIC was uniformly dispersed in the SM. However, the epoxy group peak was not observed in the SM/TGIC spectrum, confirming that the TGIC crosslinked with the soy protein, in accordance with previous research [29]. After incorporating DAS, the SM/TGIC/DAS-5 peaks were similar to those of SM/TGIC, except that the N–H peak (1458 cm^−1^) gained intensity because DAS itself contains C–H bonds. Therefore, there was no clear evidence that the DAS participated in the reaction. When introducing the HD into the SM/TGIC, the intensity of the C–N peak increased compared with that of SM/TGIC/DAS-5, indicating that the HD’s amino groups reacted with TGIC epoxy groups to form additional C–N bonds. Moreover, the amino groups in the HD facilitated the curing reaction of the epoxy TGIC. Therefore, the crosslinking density increased owing to the involvement of HD in the reaction. The crosslinking reactions of SM/TGIC/ HD are shown in Scheme 2. As the addition of HD improved from 5 to 7.5 g, the peak intensity of N–H increased, which demonstrated that the excess amino groups did not take part in the crosslinking reactions in the SM/TGIC/ HD-7.5. The excessive amino groups in the adhesives absorbed moisture more easily, which increased the water resistance. This agrees with the results of the moisture uptake study in this research.

**Figure 4:**
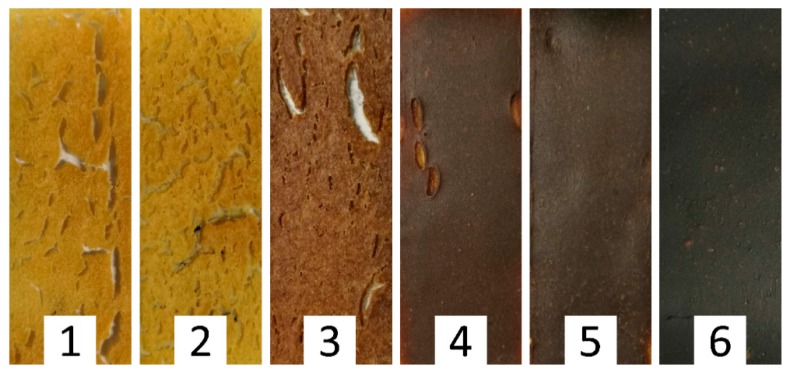
The toughness evaluation of the different adhesives 1: SM, 2: SM/TGIC, 3: SM/TGIC/DAS-5, 4: SM/TGIC/HD-2.5, 5: SM/TGIC/HD-5, and 6: SM/TGIC/HD-7.5.

Figure 4 exhibits the results of the toughness evaluation of different adhesives. Large cracks appeared in the cured SM adhesive because the hydrogen bonding interactions and molecular entanglement of the SM molecules created discontinuous fractures and poorly bonded structures during the curing process. With the introduction of TGIC, the cracks became smaller; however, the number of cracks and holes significantly increased. This was due to the adhesive’s increased brittleness, caused by the crosslinking between the SM and TGIC. For the SM/TGIC/DAS-5 adhesive, the surface became compact; however, large cracks formed on the surface, showing that the addition of DAS slightly increased the toughness of the adhesive. When the HD was added to the adhesives, the cracks were gone, and the adhesives became homogeneous and compact. Furthermore, with an increase in the HD content, the holes disappeared, and the compactness was enhanced. These results indicate that the toughness increased because the HD’s long chains enhanced the flexibility of the SM/TGIC/HD adhesives [30]; moreover, the introduction of the branched structure improved the fracture resistance and reduced the internal stress of these adhesives. This finding is consistent with reported results from previous studies [31,32]. The adhesive’s improved toughness contributed to balancing the resultant plywood’s interior force, which was beneficial to the reinforcement of the bond performance [11].

**Figure 5:**
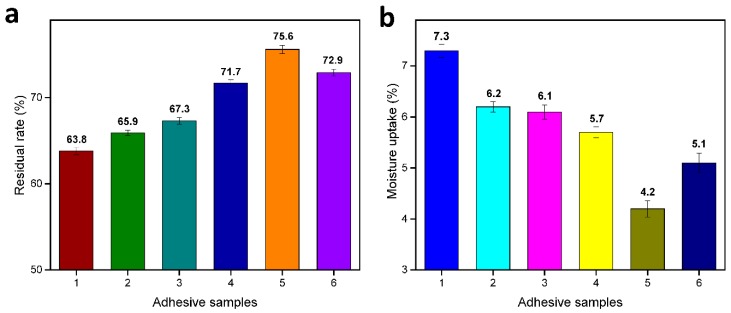
(**a**) Residual rate and (**b**) moisture uptake of the cured adhesives. 1: SM, 2: SM/TGIC, 3: SM/TGIC/DAS-5, 4: SM/TGIC/HD-2.5, 5: SM/TGIC/HD-5, and 6: SM/TGIC/HD-7.5.

The adhesive samples’ residual rates are shown in Figure 5a. The introduction of 2 g TGIC into the SM adhesive increased the residual rate from 63.8% to 65.9%. This improvement was attributed to the formation of a crosslinked structure through the reactions between TGIC and SM. The residual rate slightly improved to 67.3% with the introduction of DAS into SM/TGIC adhesive, which may be due to the low solubility of DAS at room temperature. After HD was incorporated into the SM/TGIC adhesive, the residual rate significantly improved. The highest residual rates were observed in the SM/TGIC/HD-5 adhesive (75.6%), which exceeded those of the SM and SM/TGIC/DAS-5 adhesive by 18.5% and 12.3%, respectively. Moreover, the amount of TGIC additive (2g) was 75% lower than that used in previous studies [33]. Essentially, the HD with hyper-branched amino groups, improved the degree of crosslinking, thus enhancing the corresponding adhesives’ water-resistance. However, when the HD content was up to 7.5 g, the residual rate of the adhesive reduced because the unreacted HD dissolved in water, reducing the hydrolytic stability.

Figure 5b shows that the moisture uptake of the adhesives exhibited a trend opposite to that shown by the residual rate results. By incorporating 2 g of TGIC into the SM adhesive, the moisture uptake decreased by 15.1%. These reductions were attributed to the crosslinked network formation in adhesive system, preventing water intrusion [34]. When the DAS was introduced into the SM/TGIC, the SM/TGIC/DAS-5 exhibited a negligible increase in moisture uptake compared with the SM/TGIC sample, which confirmed that the DAS did not react with the SM/TGIC adhesive. The introduction of HD into SM/TGIC adhesives can markedly decrease the water uptake of resultant adhesives. In particular, the water uptake of the SM/TGIC/HD-5 sample showed a 42.5% reduction compared with that of SM adhesive. The findings prove that HD with a hyper-branched structure participated in the reaction to improve the crosslinking density and provide a denser crosslinking structure in the adhesives, which hindered water permeability. The water uptake of the SM/TGIC/HD adhesives increased initially and then decreased with an increase in the HD content, because the unreacted HD dissolved in water, reducing the hydrolytic stability.

**Figure 6:**
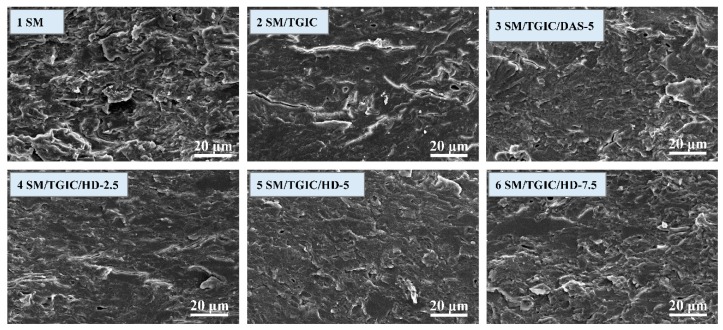
SEM images of the adhesives’ fracture surfaces.

Figure 6 shows the SEM images of the adhesives’ fracture surfaces. The entire fracture layer of the SM adhesive exhibited clutter and disorganization. A number of cracks and pores were formed by moisture evaporation during the SM adhesive’s heat curing process. These observations indicate that the adhesive layer was easily permeated by water and, therefore, exhibited poor water resistance. This result is consistent with the results from the moisture uptake analysis. For the SM/TGIC adhesive, the number of pores and cracks decreased, and the cross-section became compact because the poorly bonded SM molecules were crosslinked with the TGIC. The SM/TGIC/DAS-5 adhesive’s fracture surface was similar to that of the SM/TGIC adhesive, due to the limited reactivity of DAS. When HD was incorporated into the SM/TGIC adhesive, the adhesive’s cross-section showed a few pores and cracks but, overall, became more uniform and compact. This result is consistent with the adhesive toughness evaluation. With respect to the SM/TGIC/HD-5 adhesive, the cracks nearly disappeared from the surface. Thus, the small amount of TGIC additive (2 g) facilitated hyper-branched crosslinked structures in the adhesive system to form from the crosslinked HD.

**Figure 7:**
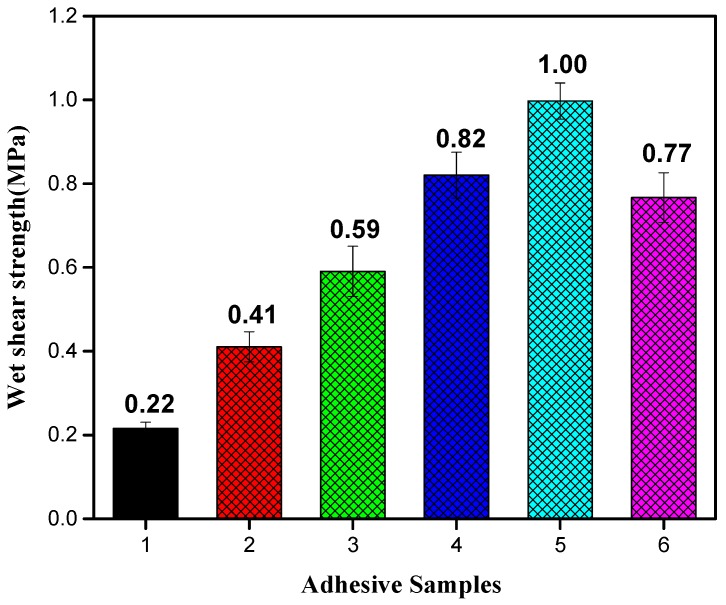
Shear strengths of the plywood prepared using various adhesives. 1: SM, 2: SM/TGIC, 3: SM/TGIC/DAS-5, 4: SM/TGIC/HD-2.5, 5: SM/TGIC/HD-5, and 6: SM/TGIC/HD-7.5.

Wet shear strengths of plywood samples prepared with resultant adhesives are shown in Figure 7. The strength of plywood prepared with SM adhesive was 0.22 MPa, not meeting the minimum strength requirement (≥0.7 MPa) for indoor use. The adhesion of SM adhesive originated from the entanglement of the protein molecules and intermolecular hydrogen bond. However, this bond is prone to cohesive failure in the presence of water. The strength of plywood prepared with SM/TGIC adhesive was 0.41 MPa, mainly benefiting from the crosslinking between TGIC and SM; however, the strength remained lower than required 0.7 MPa. After adding DAS to the SM/TGIC, the strength of resultant adhesive further increased to 0.59 MPa. Introduction of flexible DAS balanced the adhesive’s interior stress during the shear process, which led to an increase in bond strength. With the addition of HD, the bond strength of SM/TGIC/HD adhesives clearly increased and met the requirements for interior use. Notably, the TGIC addition (2 g) was reduced by 75% relative to that in previous studies [35]. The strength of plywood prepared with SM/TGIC/HD-5 adhesive improved up to 1.00 MPa, reflecting increases of 354.5% and 69.5% relative to that of the SM and SM/TGIC/DAS-5 adhesives, respectively. In these systems, the crosslinking density of adhesives improved with the incorporation of HD. The crosslinked HD and SM afforded dense three-dimensional networks to enhance the water resistance. However, when the HD addition was up to 7.5 g, the resultant strength reduced to 0.77 MPa. This reduction in shear strength suggests that the excess HD absorbed water and caused a decrease in strength, which is in agreement with the findings from the above moisture uptake analysis.

Notably, this adhesive with a hyper-branched crosslinking structure exhibits high strength, toughness, water resistance, and it has been successfully applied in the pilot-scale industrial production and the resultant plywood can be used indoor with enough bond strength.

## 4. Conclusions

Hyper-branched starch HD, a novel long-chain starch with hyper-branched structure, was synthesized through graft copolymerization of HBPA onto DAS. The results from calculations of ^1^H NMR analysis showed that the grafting rate of HD reached 75.8%. Due to its hyper-branched structure, the HD featured a higher thermal stability. The residual rate of SM/TGIC/HD-5 adhesive increased by 18.5% when 5 g of HD was incorporated into the SM/TGIC adhesive. In addition, the wet shear strength of plywood prepared with the proposed adhesive increased by 354.5% to 1.00 MPa, relative to that of the plywood using the SM-only adhesive, meeting the strength requirement for indoor use. The superior properties of SM/TGIC/HD-5 adhesive are attributed the following primary factors: (1) The TGIC reacted with the soy proteins and HD to form a hyper-branched crosslinked structure that increased the adhesive’s crosslinking density and water-resistance; (2) this hyper-branched crosslinking structure created a smooth/dense cross-section, which reduced the moisture uptake by 42.5% relative to that of the SM-only adhesive; (3) because HD is inherently flexible, the resultant adhesive’s toughness improved. Thus, when the HD adhesive was applied to the plywood, it helped maintain balance in the plywood’s interior force, which further improved the adhesive’s bond performance. In addition, forming a hyper-branched structure in the adhesive effectively reduced the necessary TGIC dosage. Only 2 g of TGIC was used to synthesize this superior adhesive; 75% less than the amount of TGIC used in previous studies, which is also applicable to the petroleum-based crosslinking agents. Thus, the resultant adhesive is nontoxic and cost effective. Moreover, all the experimental evidence indicates that the adhesive features high water resistance and excellent mechanical properties, and it has been successfully applied in the pilot-scale industrial production and the resultant plywood met the interior-use requirements.

## Figures and Tables

**Table 1:** Grafting rate data.

Integral area (*A*)				Molar mass
*A*_1_ = 53.43	*A*_2_ = 25.60	*A*_0-1_ = 8.14	*A*_0-2_ = 10.20	*M*_0_ = 210.09

## Supplementary Materials

The following are available online at https://www.mdpi.com/2073-4360/11/8/1352/s1, Figure S1: the GPC chromatograms of dialdehyde starch (DAS), Table S1: different adhesive formulations, Scheme S1: the synthesis procedure of hyper-branched polyamide (HBPA), Scheme S2: aldehyde release mechanism of DAS.
