# Supplementary material for: A Tough, Water-Resistant, High Bond Strength Adhesive Derived from Soybean Meal and Flexible Hyper-Branched Aminated Starch
Source: Polymers (Basel). 2019 Aug 14;11(8):1352. doi: 10.3390/polym11081352 (PMC6722635; doi:10.3390/polym11081352)
Supplement: Supplementary file 1 [file polymers-11-01352-s001.pdf]

Supporting information

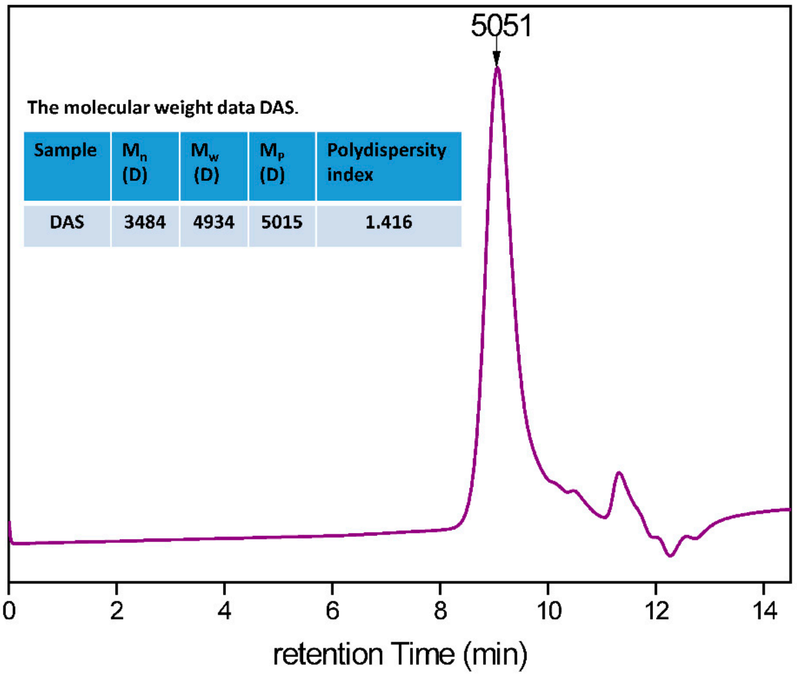

Figure S1. The GPC chromatograms of dialdehyde starch (DAS).

Table S1. Different adhesive formulations.

Number	Sample	Adhesive formulation
1	SM	SM (30 g); deionized water (70 g)
2	SM/TGIC	SM (30 g); deionized water (70 g); TGIC (2 g)
3	SM/TGIC/DAS-5	SM (30 g); deionized water (70 g); TGIC (2g); DAS (5 g)
4	SM/TGIC/HD-2.5	SM (30 g); deionized water (70 g); TGIC (2 g); HD (2.5 g)
5	SM/TGIC/HD-5	SM (30 g); deionized water (70 g); TGIC (2 g); HD (5 g)
6	SM/TGIC/HD-7.5	SM (30 g); deionized water (70 g); TGIC (2 g); HD (7.5 g)

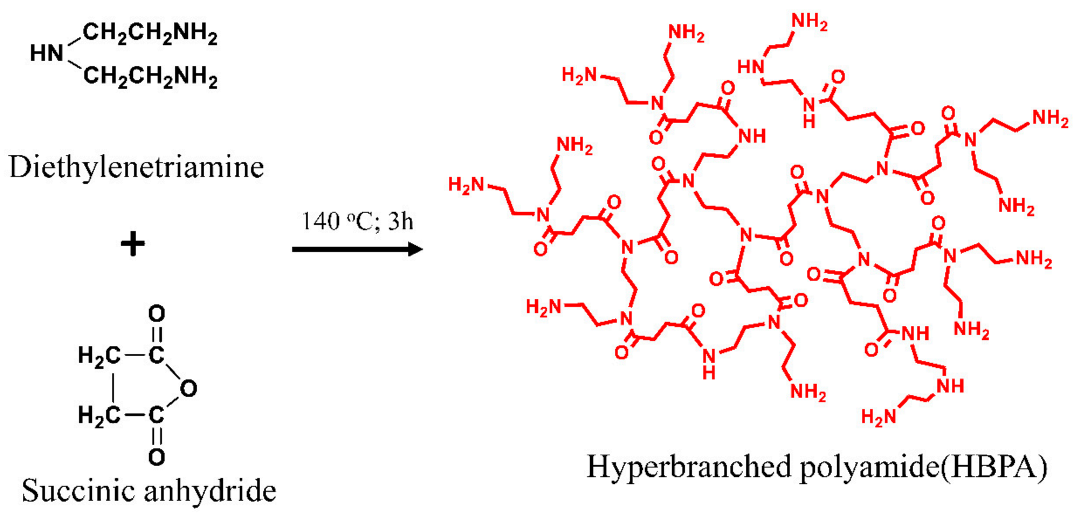

Scheme S1. The synthesis procedure of hyper-branched polyamide (HBPA).

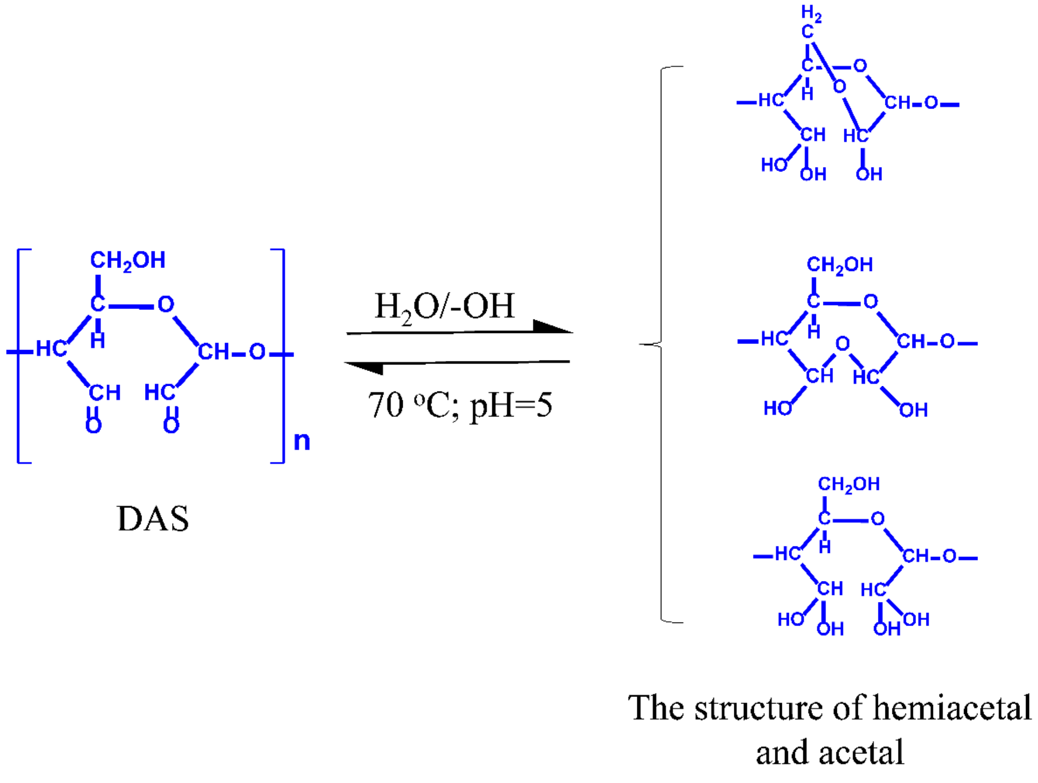

Scheme S2. Aldehyde release mechanism of DAS.
